# CECs and IL-8 Have Prognostic and Predictive Utility in Patients with Recurrent Platinum-Sensitive Ovarian Cancer: Biomarker Correlates from the Randomized Phase-2 Trial of Olaparib and Cediranib Compared with Olaparib in Recurrent Platinum-Sensitive Ovarian Cancer

**DOI:** 10.3389/fonc.2015.00123

**Published:** 2015-06-01

**Authors:** Jung-Min Lee, Jane B. Trepel, Peter Choyke, Liang Cao, Tristan Sissung, Nicole Houston, Minshu Yu, William D. Figg, Ismail Baris Turkbey, Seth M. Steinberg, Min-Jung Lee, S. Percy Ivy, Joyce F. Liu, Ursula A. Matulonis, Elise C. Kohn

**Affiliations:** ^1^Center for Cancer Research, Bethesda, MD, USA; ^2^Cancer Therapy Evaluation Program, National Cancer Institute, Bethesda, MD, USA; ^3^Dana-Farber Cancer Institute, Boston, MA, USA

**Keywords:** CEC, IL-8, biomarkers, olaparib, cediranib, ovarian cancer

## Abstract

**Objective:**

Olaparib (O), a polyADPribose polymerase (PARP) inhibitor, and cediranib (C), a VEGF receptor (VEGFR)1–3 inhibitor together had greater activity than O alone in women with recurrent platinum-sensitive ovarian cancer (OvCa). The objective of this study is to identify potential lead biomarker candidates for response to O + C in the setting of a multi-institutional phase II study of O with and without C in recurrent platinum-sensitive OvCa.

**Methods:**

A self-selected group of patients participated in a prospectively planned exploratory biomarker substudy of the randomized phase II study of O versus O + C. Whole blood for peripheral blood mononuclear cell (PBMC) and plasma isolation was collected prior to and on day 3 of treatment. Quantitation of circulating endothelial cells (CEC), IL-6, IL-8, VEGF, and soluble VEGFR-2 plasma concentrations, and polyADPribose (PAR) incorporation were performed. Single nucleotide polymorphism analysis of *XRCC1* 280H, R194W, and Q399R was done. Dynamic contrast-enhanced-magnetic resonance imaging (DCE-MRI) was performed at baseline and day 3 of treatment. Parameter changes were compared between the two arms using an exact Wilcoxon rank sum test. Kaplan–Meier and log-rank tests were used to examine survival outcome.

**Results:**

Thirteen patients elected to participate in the translational substudy, seven patients on O and six patients on O + C. Patients on O + C had a greater decrease in IL-8 concentration and larger CEC fold increase compared with those on O alone (*p* = 0.026, *p* = 0.032). The fold increase in CEC on day 3 was associated with duration of progression-free survival (PFS) (*R*^2^ = 0.77, 95% CI 0.55–0.97, *p* < 0.001). IL-8 post-pretreatment changes correlate with PFS (*p* = 0.028). *XRCC1* DNA polymorphisms were not related to PFS. All patients had reduction in PAR incorporation, and all except one had reduction in vascular flow on DCE-MRI.

**Conclusion:**

Our exploratory correlative studies indicate that CEC and IL-8 changes may be predictive for response to O + C and prognostic in recurrent platinum-sensitive OvCa, requiring prospective validation.

## Introduction

Ovarian cancer (OvCa) accounts for the majority of gynecologic cancer deaths in the United States (1). Most women with OvCa present with advanced disease, and recurrence is nearly universal leading to incurable disease and limited treatment options (2, 3). The optimal way to incorporate new agents into OvCa treatment and when to initiate these agents remains a question. Women whose disease progresses more than 6 months after exposure to platinum agents tend to respond better to subsequent interventions than those with platinum-resistant or refractory disease, making them another important cohort in whom to investigate new directions. OvCa is an angiogenic cancer and activity has been shown with inhibitors of the VEGF/VEGF receptor (VEGFR) axis (2, 4–8). The recognition that OvCa may be driven by disordered DNA damage repair led to the development and recent approval of polyADPribose polymerase (PARP) inhibition for therapeutic benefit. Therefore, inhibition of both DNA damage repair and angiogenesis pathways would be an important direction to be examined.

Preclinical studies demonstrate potential interaction between angiogenesis and DNA damage repair pathways. γH2AX, a marker of the DNA damage repair response, is necessary for endothelial cell proliferation under hypoxia and hypoxia-driven neovascularization *in vivo* (9). It has been shown that hypoxia leads to downregulation of BRCA1 and RAD51, making hypoxic lung cancer cells more sensitive to PARP inhibitors (PARPi) (10). PARP1 inhibition also increases VEGFR-2 phosphorylation and subsequent activation of endothelial cell survival in human umbilical vein endothelial cells, an effect, which was reversed by a VEGFR-2 inhibitor (11). Our preliminary work showed that the combination of olaparib and cediranib inhibited invasion of OvCa cells, in a more than additive fashion. Invasion was significantly decreased in pretreated OvCa cell lines, CAOV3 and OVCAR8, exposed to concentrations attainable in patients, cediranib 50 nM or olaparib 10 μM, or the combination (*p* < 0.0001 for all treatments). Additionally, olaparib and cediranib inhibited microvascular endothelial cell tube formation on Matrigel at concentrations well below those clinically attainable in patients. Cumulative tube length was significantly reduced when endothelial cells were exposed to cediranib 5 nM or olaparib 100 nM (all *p* < 0.05). The combination of olaparib and cediranib resulted in greater inhibition of tube formation than monotherapy (*p* < 0.0001, Kim et al., unpublished data). These preclinical data suggest new strategies for novel combination therapies in recurrent OvCa. We hypothesized that introduction of angiogenesis inhibitors and PARPi will have activity and examination of the biology should lead to potential predictive biomarkers (7, 12, 13).

PolyADPribose polymerase inhibitors are a novel class of drugs designed to compete with NAD^+^ for the substrate binding site of PARP, preventing DNA single strand break repair process through the base excision repair pathway (14). Another mechanism of PARPi includes trapping of PARP1 and PARP2 while in complex with damaged DNA, resulting in cytotoxic consequences (15). Trapped PARP prevents its availability for repair function and secondarily causes replication and transcription fork blockade, and subsequent DNA breakage. Olaparib, a PARPi, was approved recently by the US Food and Drug Administration for use in advanced OvCa patients bearing deleterious germline *BRCA1* and *BRCA2* mutations (gBRCAm); clinical activity has also been reported in sporadic OvCa (14, 16). Cediranib is a small-molecule tyrosine kinase inhibitor of VEGFR1–3 and c-kit with modest single agent activity in recurrent OvCa (8, 17). In the ICON 6 study, cediranib combined with platinum and paclitaxel standard chemotherapy followed by maintenance cediranib treatment significantly prolonged progression-free survival (PFS) and overall survival (OS) when administered to women with first recurrence of platinum-sensitive OvCa (17).

We recently reported a randomized phase-2 multi-institutional study of olaparib capsules with or without cediranib for recurrent platinum-sensitive OvCa, showing the combination improved PFS (17.7 versus 9 months, *p* = 0.005) and response rate (80 versus 48%, *p* = 0.002) (13). Prospectively planned exploratory biomarker endpoints were included. The aim of this translational study is to identify potential predictive biomarker candidates for olaparib and cediranib by assessment of vascular and DNA repair endpoints within the multi-institutional phase 2 study of olaparib and cediranib.

## Patients and Methods

### Patients

**Figure 1:**
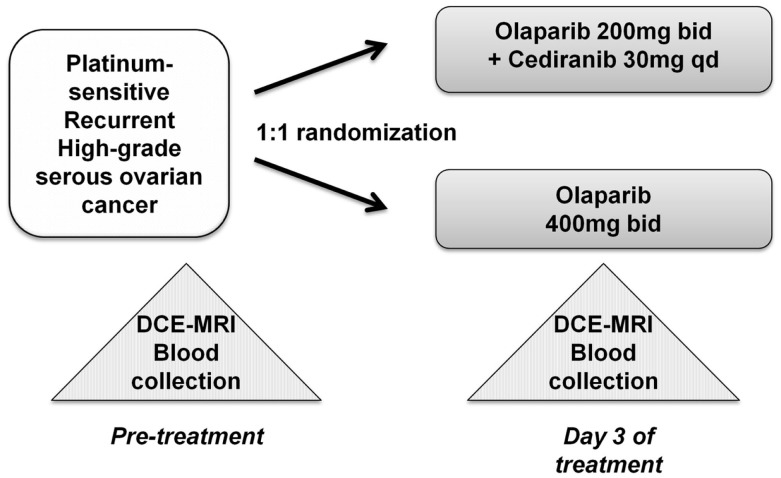
**Treatment schema and correlative studies**. Peripheral blood mononuclear cell (PBMC), whole blood DNA, and plasma samples were collected prior to initiation of the study drug(s), and all patients underwent DCE-MRI imaging. Follow-up sampling was done on day 3 of treatment.

The randomized study with the internal translational substudy was approved by the institutional review boards of the participating sites, and written informed consent was obtained from participating patients; clinical study details including drug administration, safety, adverse events, and tumor response have been reported and the schema for treatment is shown in Figure 1 (13). The patients who participated in the translational substudies underwent dynamic contrast-enhanced-magnetic resonance imaging (DCE)-MRI and blood collection prior to and on day 3 of treatment. Study treatment, imaging, and blood collection occurred at the Center for Cancer Research, NCI after which the patients continued care at their primary investigational site.

### Quantitation of circulating endothelial cells and circulating endothelial progenitor cells

Blood was collected in citrated CPT cell preparation tubes (BD Vacutainer, Franklin Lakes, NJ, USA). PBMCs were isolated, aliquoted, and viably frozen within 2 h of collection, and stored at −80°C until use. Circulating endothelial cells (CEC) and circulating endothelial progenitor cells (CEP) analyses were performed using an MACSQuant flow cytometer (Miltenyi Biotec, Bergisch Gladbach, Germany); a minimum of 1 × 10^5^ cells were acquired for each analysis. CECs were defined as negative for the hematopoietic marker CD45 (leukocyte common antigen), positive for the endothelial markers CD31 and CD146, and negative for the progenitor marker CD133. CEPs were defined as the CD45-/CD31+/CD146-/CD133+ population (18, 19). Viability was defined by the absence of 7-aminoactinomycin D (7-AAD) staining, and analysis was restricted to nucleated cells by gating on Hoechst 33342-positive cells (20, 21). Data were analyzed using FlowJo software (FlowJo LLC, Ashland, OR, USA).

### Cytokine analysis

Plasma samples were collected in K2EDTA tubes (BD Vacutainer) and were processed within 2 h of collection. After centrifugation, the samples were aliquoted, immediately frozen, and stored in liquid nitrogen until use. Quantitative analysis of circulating plasma VEGF, IL-6, IL-8, and soluble VEGFR-2 were performed using analytically validated custom V-PLEX assay plates on an electrochemiluminescence platform according to the manufacturer’s instructions (Meso Scale Discovery, Gaithersburg, MD, USA) (22). The concentrations of the cytokines were determined with recombinant standards and expressed as picograms per milliliter, as reported (23).

### Measurements of PAR incorporation, and isolation of DNA for single nucleotide polymorphism analysis

Peripheral blood mononuclear cell DNA PARylation was measured using a commercial immunoassay according to the manufacturer’s instructions (Trevigen, Gaithersburg, MD, USA) (24). PBMC DNA was isolated for polymorphism analysis of *XRCC1* 280H, R194W, and Q399R using a commercial DNA purification kit (Qiagen, Germantown, MD, USA), as reported (25).

### DCE-MRI functional imaging

Dynamic contrast-enhanced-magnetic resonance imaging (DCE-MRI) was performed to assess changes in vascular permeability (*K*_trans_) and perfusion (*K*_ep_) (26). MRI data were analyzed using a two-compartment model based on the general kinetic (GKM) Kety model using commercial software (iCAD, Nashua, NH, USA) as reported (27, 28). The GKM model analysis was done with an Interactive Data Language-based (Research Systems Inc.) research tool (Cine Tool; GE Healthcare). Manual region-of-interest measurements were obtained from each slice of the target lesion. The GKM model produces three parameters: *K*_ep_, the reverse contrast transfer rate; *K*_trans_, the forward contrast transfer rate; and *V*_e_ the extravascular fraction. Baseline and day 3 of treatment *K*_ep_, *K*_trans_, and *V*_e_ values were obtained.

### Statistical analysis

Parameter changes were compared between the two arms using an exact Wilcoxon rank sum test. The probability of PFS as a function of time was estimated using the Kaplan–Meier method, with a log-rank test to determine the significance of the differences. The cut-off date for PFS and endpoint analysis of this subset was March 31, 2014 consistent with the original study. All *p*-values are two-tailed and reported without adjustment for multiple comparisons due to the small substudy cohort.

## Results

### Patients

This substudy reports on 13 self-selected participants of the multi-institutional randomized phase-2 study of recurrent platinum-sensitive OvCa patients who were treated with olaparib capsules (200 mg twice a day) and cediranib (30 mg daily) or olaparib capsules alone (400 mg twice a day) until disease progression (13). Participating patient details are shown in Table 1, demonstrating their representation of the full patient cohort in terms of age, treatment arm distribution, and clinical outcome. Not all patients could undergo imaging within the required time frame; Table 1 also includes the number and distribution of patients within each of the substudy elements.

**Table 1:** **Clinical characteristics (*n* = 13)**.

	Olaparib	Olaparib + cediranib
	7 patients	6 patients
Age: median 53 (range 32–70)	53 (32–61)	57 (53–70)
Number of prior lines of therapy	1: 6 patients	1: 3 patients
	2: 1 patients	2: 2 patients
		3: 1 patients
Response rate	57%	83%
Best response	PR: 4	PR: 5
	SD >4 months: 3	SD >4 months: 1
PFS*, median	11.2 months (3.6–16.8)	13.8 months (7.5–22.2+)
*BRCA*1 or *BRCA*2 mutational status	Mutated: 4 – or unknown: 3	Mutated: 3 – or unknown: 3
Paired correlative studies
Cytokine^a^	6 patients	6 patients
PAR incorporation^b^	6 patients	5 patients
CEC/CEP^c^	5 patients	5 patients
DCE-MRI^d^	4 patients	6 patients

*p = 0.60 for overall comparison, eight PFS events were observed, four patients on each arm at the time of data cut-off (March 31, 2014).

*^a^Cytokine samples from one patient were missing*.

*^b^Two patients had no optimal viable cells from the frozen samples for PAR analysis*.

*^c^Three patients had no optimal viable cells from the frozen samples for CEC/CEP analysis*.

*^d^Three patients were unable to tolerate DCE-MRIs*.

### Quantitation of CEC and CEP

**Figure 2:**
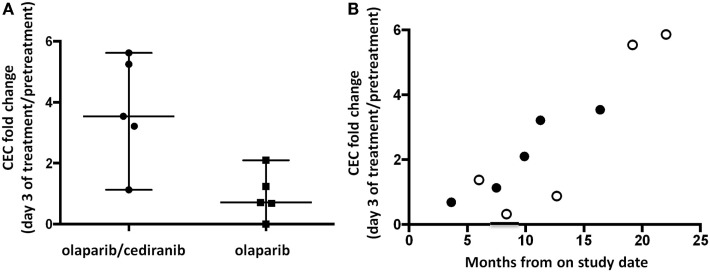
**Circulating endothelial cells (CEC) and circulating endothelial progenitor cells (CEP)**. **(A)** Patients receiving olaparib/cediranib had a larger fold increase in CEC compared to olaparib alone during treatment (*p* = 0.032). **(B)** The fold increase in CEC on day 3 was associated with PFS duration in all patients (*R*^2^ = 0.77, 95% CI 0.55–0.97, *p* < 0.001) when reporting values on a patient-by-patient basis. The open dots represent patients whose times at risk were censored on the date of their last contact and were last reported alive and progression-free. This observed pattern may not retain the linear relationship as time passes.

It has been reported that inhibition of angiogenesis induces a feedback response with induction of angiogenic precursors (29–31). Patients receiving both agents had a median 3.5-fold increase in CEC compared to 0.7 for patients on single agent olaparib (*p* = 0.032, Figure 2A). CEC fold increase pretreatment to day 3 was associated with time to progression or follow-up without progression when viewed on a patient-by-patient basis using available follow-up data (*R*^2^ = 0.77, 95% CI 0.55–0.97, *p* < 0.001; Figure 2B). Baseline values of CEC and CEP were not associated with PFS. CEP fold change was not significantly different between the two arms.

### Cytokine analysis

**Figure 3:**
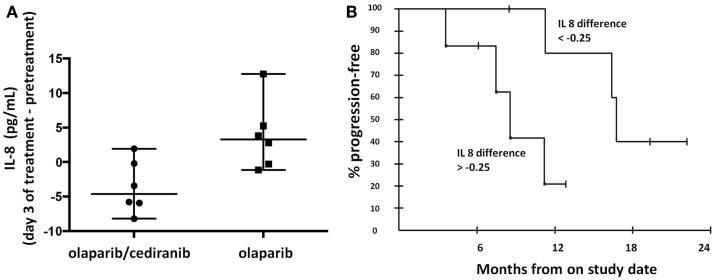
**Proangiogenic cytokines**. **(A)** Patients on olaparib/cediranib had a greater change in circulating IL-8 concentration with treatment than patients on olaparib alone (*p* = 0.026). **(B)** Patients with greater IL-8 change [below the median IL-8 difference (−0.25)] was associated with longer PFS (*p* = 0.028). The 6-month PFS probability for the six patients with changes above the median was 83.3% (95% CI 43.6–97.0), while for the group with the changes below the median, it was 100% (95% CI 54.1–100). At 12 months, the respective probabilities of PFS were 20.8% (95% CI 3.8–63.6) and 80.0% (95% CI 37.6–96.4).

Circulating cytokines have been proposed as potential biomarkers of response to anti-angiogenics (32, 33). Our findings showed that greater decreases in circulating IL-8 concentrations in patients treated with olaparib and cediranib than with olaparib alone (*p* = 0.026, Figure 3A). The median IL-8 difference with the combination of olaparib and cediranib was −0.25. The patients with greater decrease than median IL-8 difference was associated with longer PFS (*p* = 0.028, Figure 3B). Median values of pretreatment IL-8 concentration were not significantly different between the two arms. Pretreatment values of other cytokines including circulating plasma VEGF, soluble VEGFR-2, and IL-6 examined did not correlate with PFS and was not different between two arms. Values of other cytokines did not significantly change after treatment in either arm.

### Measurements of PAR incorporation and polymorphism analysis

**Figure 4:**
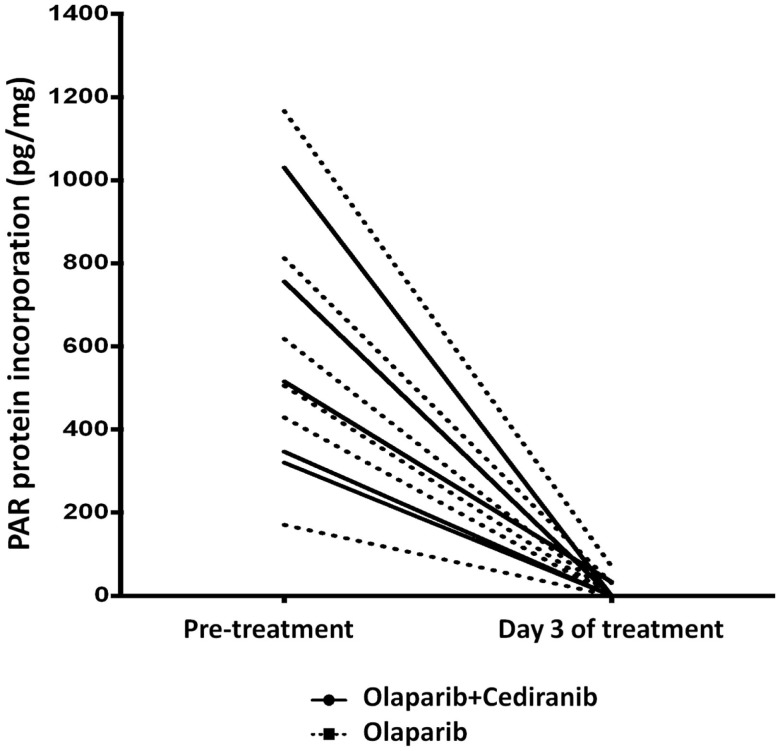
**PAR incorporation**. Marked reduction in PAR was seen in all patients treated with olaparib-based therapy. Solid line represents patients treated with olaparib/cediranib and dotted line represents patients treated with olaparib alone.

PolyADPribose polymerase inhibitors activity was measured by polyADPribose (PAR) incorporation into PBMC DNA. Reduction in PBMC PAR incorporation with treatment was observed in both arms, indicating the lower dose of olaparib results in full PAR inhibition and that addition of cediranib does not diminish that olaparib function (Figure 4). We also examined whether *XRCC1* DNA polymorphisms correlate with clinical response to PARPi and no significant associations with PFS were observed.

### Dynamic contrast-enhanced-magnetic resonance imaging

**Figure 5:**
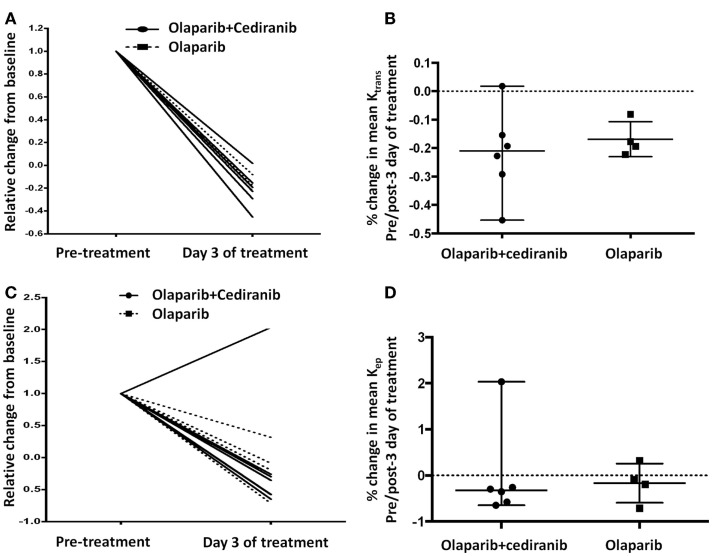
**DCE-MRI**. **(A,B)** All patients had reduction of *K*_trans_ without statistically significant differences between arms (*p* = 0.61). Solid line represents patients treated with olaparib/cediranib and dot line represents patients treated with olaparib alone. **(C,D)** All but one patient had reduction of *K*_ep_ after treatment without significant difference between arms (*p* = 0.61). Solid line represents patients treated with olaparib/cediranib and dotted line represents patients treated with olaparib alone.

Permeability and perfusion calculations by DCE-MRI have been used to characterize tumor vasculature changes in response to the VEGF/VEGFR axis inhibitors (34). In this study, all patients except one had a reduction with treatment in *K*_trans_ and *K*_ep_ independent of treatment arm; increase of *K*_ep_ on day 3 of treatment in one patient was likely due to suboptimal contrast injection timing (Figures 5A,C). No significant differences were observed between the two treatment arms (Figures 5B,D). All patients had at least stable disease at the first Response Evaluation Criteria in Solid Tumors (RECIST) evaluation after two cycles of treatment, indicating potential benefit but no correlation with the duration of response.

## Discussion

No validated biomarkers predictive of anti-angiogenic response have been defined to date. We hypothesized that olaparib and cediranib in combination would yield greater vascular injury and clinical activity than olaparib alone in recurrent OvCa. Our exploratory translational studies suggest that CEC fold change and IL-8 change on day 3 of treatment may represent biomarkers predictive for response to the olaparib and cediranib combination.

The presence of CEC has been recognized as a potential biomarker of vascular damage (19, 35). CEPs have been shown to infiltrate human tumors and give rise to tumor neovasculature, whereas mature CECs derive from mature vasculature (36). Thus, inhibition of the VEGF pathway can attenuate bone marrow-derived CEPs mobilized by VEGF (37) and can trigger an increase in circulating mature CECs, reflecting sloughing of fragile mature endothelium, potentially from tumor vasculature (38, 39). The number of CECs was reported significantly higher in patients with metastatic cancer compared with healthy donors (40). Elevated numbers of CEC, reflecting vascular endothelium perturbation by anti-angiogenics, have been described in lymphoma, and solid tumors including OvCa (18, 19, 35). An increase in CEC numbers after 6 weeks of treatment correlated with >75% PSA decline in metastatic castrate-resistant prostate cancer treated with bevacizumab and thalidomide, in addition to docetaxel and prednisone (35). Our findings support the hypothesis that greater vascular injury may correlate with clinical response and present an opportunity to develop a predictive biomarker to this novel combination.

Most of the inhibitors of angiogenesis are associated with altered regulation of proangiogenic and proinflammatory cytokines. Plasma cytokine and angiokine concentrations have been examined in advanced cancers (32, 41–43). Economopoulou and colleagues demonstrated that functional γH2AX is required for cell response to aggravated hypoxia indicating a need for DNA repair in angiogenesis (9). Thus, it was posited that there would be greater differences in the pharmacodynamic cytokine response to the combination of olaparib and cediranib. Our findings indicate that IL-8 changes correlated with PFS. IL-8 plays an important role in tumor growth and metastasis, and is associated with tumor burden in melanoma, renal cell cancer, and hepatocellular carcinoma *in vivo* models and with worse prognosis in advanced solid tumors including OvCa (44–46). In our study, IL-6, circulating VEGF, and soluble VEGFR-2 had no significant change after treatment and were not different between two arms, although they have been reported as prognostic biomarkers in OvCa (47, 48). It is possible that no difference was observed due to a small sample size and premature sample collection times to assess the dynamic changes in additional cytokine biomarkers during treatment. Further studies will be needed to consider more comprehensive cytokine panels and various time points of sample collection.

Incorporation of polyADP ribose moieties at sites of double-stranded DNA breaks is a signal to the repair machinery to initiate repair, though it is not a marker of repair *per se* (49). It was initially hypothesized that modulation of PAR incorporation by PARPi would be both proof of mechanism and could be used to predict outcome. In our current study, we prospectively planned to measure PAR incorporation into PBMC DNA. Proof of mechanism was shown in an early phase 0 study of ABT-888 (veliparib) but we and others have now shown that this assay in PBMCs does not predict clinical benefit (25, 49, 50).

Functional tumor imaging with DCE-MRI is capable of evaluating changes in vascularity and quality of index lesions (27). Our results demonstrated vascular flow changes after treatment but did not correlate with clinical outcome. It has been shown that the changes of DCE-MRI variables at baseline and at day 28 of cediranib monotherapy were significantly associated with PFS in metastatic castrate-resistant prostate cancer patients (51). Chase et al. reported relative blood flow (RBF) measured by DCE-MRI pre-cycle 1 and pre-cycle 4 of bevacizumab in 13 women with recurrent OvCa. RBF remained stable for the majority of the cases (median change −0.21) and was not related to PFS or OS (34). DCE-MRI may provide early indications of treatment effect even before changes in size can be perceived on CT, requiring further exploration with optimal sample size and time points (52).

The combination of olaparib and cediranib has marked clinical activity in women with recurrent platinum-sensitive OvCa. Our exploratory translational studies suggest that olaparib and cediranib caused vascular injury and decrease in angiogenesis indicated by an increase of CEC and decrease in IL-8. These findings provide insights into the biological effects and represent potential predictive biomarkers for response to the combination. Consideration of additional time points and monitoring in both PBMCs and tissue samples may further improve the overall clinical utility of these endpoints. Further clinical exploration of these biomarkers is warranted in patients with OvCa treated with olaparib and/or cediranib.

## Conflict of Interest Statement

The authors declare that the research was conducted in the absence of any commercial or financial relationships that could be construed as a potential conflict of interest.
